# Determinants of COVID-19 skepticism and SARS-CoV-2 vaccine hesitancy: findings from a national population survey of U.S. adults

**DOI:** 10.1186/s12889-022-13477-2

**Published:** 2022-05-25

**Authors:** Jeff Levin, Matt Bradshaw

**Affiliations:** Baylor University, One Bear Place # 97236, Waco, TX 76798 USA

**Keywords:** COVID-19,, SARS-CoV-2,, Vaccine hesitancy,, Immunization,, Politics,, Religion,, Health policy

## Abstract

**Background:**

The enduring presence of COVID-19 skepticism and SARS-CoV-2 vaccine hesitancy is an ongoing impediment to the global response effort to the current pandemic. This study seeks to identify determinants of skepticism and vaccine hesitancy in U.S. adults.

**Methods:**

Data are from the Values and Beliefs of the American Public Survey, conducted in 2021 by the Gallup Organization in conjunction with Baylor University. The survey used stratified random probability sampling of the U.S. adult population (*N* = 1222). Outcome measures were respective single items assessing COVID-19 skepticism and SARS-CoV-2 vaccine hesitancy. Exposure variables included political, religious, and sociodemographic indicators, and moderators assessed personal history of COVID-19 and losing a relative or close friend to COVID-19.

**Results:**

Skepticism and vaccine hesitancy were strongly associated with conservative and Republican political preference and conservative religious beliefs, and less so with socioeconomic status. Personal experience with COVID-19 did not mitigate the effect of politics on skepticism and barely reduced the odds for hesitancy. Results confirm that attitudes toward COVID-19 are politically and religiously conditioned, and are especially a product of conservative political preference.

**Conclusion:**

Skepticism about COVID-19 and hesitancy regarding SARS-CoV-2 vaccination are highest among the political and religious right. Efforts to increase immunization through public education may be inadequate; resistance appears ideological. Other solutions may need to be considered, which risk widespread pushback both politically and religiously motivated.

## Background

Following a robust rollout at the end of 2020, the introduction of the various SARS-CoV-2 vaccines was met with enthusiastic adoption in the U.S., and by mid-January, 2021, the basic reproduction number had fallen below 1.0 in over 40 states [1], indicating a slowing of transmission and raising the possibility of eventually attaining herd immunity [2]. Epidemiologists and public health scientists know what happened next: early successes in vaccinating the general population led to declines in COVID-19 incidence and fatalities [3], but, then, perhaps unexpectedly, things hit a wall. By April, 2021, the daily count of vaccine doses administered to the U.S. population, which to that point had been ascending quickly, essentially went over a cliff and, since July, 2021, has risen again only modestly [4]. This hesitancy to get vaccinated, fueled in part by misperceptions reinforced by media coverage, has led to multiple adverse social, political, and public health outcomes. Most notable has been the presence of a large enough “space” of unvaccinated individuals to enable highly transmissible variants to adapt, evolve, emerge, and spread throughout the unvaccinated population and into a vaccinated population beginning to experience waning immunity. As of this writing, the U.S. has finally emerged from a healthcare crisis exceeding in magnitude the experience with the first wave in 2020 [5], but once-optimistic talk of herd immunity has mostly been relegated to the back burner [6]. Ongoing surveillance and predictive models suggest that another outbreak of cases may be on the horizon later in 2022 [7]. Throughout 2021 and 2022, the public health sector has been struggling to identify the large pockets of vaccine hesitancy and to answer a simple question: who are these individuals?

Anecdotally, it is widely held that SARS-CoV-2 vaccine-hesitant individuals in the U.S. are mostly at the far rightward end of the political and religious spectrum. The provenance of the broader and longer-standing antivaccine movement, however, suggests something more complex [8]. The contemporary spread of the antivaccine ideology is owed in large part to Andrew Wakefield, a now delicensed British physician who famously authored a study suggesting that the MMR vaccine was responsible for autism, a study later exposed as fraudulent [9]. After an investigation, *The Lancet* retracted the paper, ten of the co-authors disavowed the paper, and Wakefield left the U.K. in disgrace, settling in Austin, Texas, but not before causing what has been called “the most damaging medical hoax of the last 100 years” [10]. Once in the U.S., his discredited findings fueled something of a social movement that soon gained traction on both coasts and in certain urban centers, via adoption by people espousing progressive attitudes toward politics, diet and health, animal rights, environmentalism, and spirituality and who sought nonmedical exemptions for their children’s required vaccinations [11]. These views resemble those of the earliest antivaccinators nearly 200 years ago [12].

Within a few years of the autism controversy, due in part to the debunked study’s endorsement by several highly visible televangelists, notably the principals of Kenneth Copeland Ministries, the antivaccine movement fanned outward to politically conservative evangelical, Pentecostal, and fundamentalist Christians throughout the U.S. [13], and more recently to Somali Muslim immigrants in Minnesota [14] and ultra-Orthodox Jews in New York [15]. While political and religious opposites, both of these population groups—secular progressives and religious conservatives—seem to be implicated in SARS-CoV-2 vaccine hesitancy, according to media reports. Yet despite these much commented upon observations, there has not yet been empirical confirmation through representative national probability-sample data. This will continue to be a significant issue for the U.S. White House COVID-19 Response Team and its technical advisors helping to craft the nation’s strategy to reach the still substantial vaccine-hesitant segment of the population [16], both in the U.S. and globally [17].

Aside from vaccine hesitancy, a related challenge emerged months before a vaccine was made available—indeed almost as soon as the first COVID-19 cases appeared and well before the virus’ genome had even been sequenced. That issue has been skepticism or disbelief that the coronavirus is as pathogenic or virulent as advertised, or is even real [18]. Public health professionals are likely familiar with the tales of alleged conspiracies [19]—some perhaps semi-plausible early on (e.g., SARS-CoV-2 is a lab-weaponized pathogen), others quite ridiculous (e.g., COVID-19 is not a viral disease but is due to radiation from 5G towers) [20]. Vaccine hesitancy has spawned its own bizarre conspiracies [21], such as Bill Gates installing microchips or magnetic nanobots in the syringes for purposes of government surveillance, and its own disinformation [22], such as the vaccine not really being a vaccine, or the vaccine causing AIDS. Over two years into the pandemic, at the time of this writing, skepticism and hesitancy are twin impediments that, together, have created enough suspicion to have partly derailed the vaccine rollout and to have contributed to depressed rates of immunization throughout the U.S. [23]. This in turn may present a substantial impediment to finally reducing the incidence of COVID-19 cases to a manageable level, even to moderate endemicity.

Vaccine hesitancy has been shown to have historical, political, and sociocultural antecedents [24]. Clusters of unvaccinated individuals exist within every country, even those with high overall rates of immunization [25]. Early in the COVID-19 pandemic, national data indicated that 31.6% of the U.S. population was unsure about receiving a vaccine, and 10.8% stated that they would refuse [26]. These numbers suggest that months before a vaccine had even been developed, herd immunity may have been a pipe dream. Moreover, there are other downstream effects: SARS-CoV-2 vaccine hesitancy has already contributed to sudden and substantial declines in routine childhood vaccinations [27, 28], and American and Canadian veterinarians now report an impact on pet owners’ hesitancy to vaccinate their dogs and cats [29]. As was noted several years ago, “Determinants of vaccine hesitancy are complex and context-specific—varying across time, place and vaccines” [30]. In other words, the present situation may be *sui generis* and present a unique set of circumstances with unique antecedents, although, as with vaccine hesitancy in general, political, religious, and socioeconomic determinants are typically observed [31].

The present study seeks to provide some confirmation or clarification as to the identity of those U.S. adults who endorse COVID-19 skepticism and/or SARS-CoV-2 vaccine hesitancy. Based on prior reports of political correlates of misperceptions about COVID-19 [32, 33], along with decades of exposés of the antivaccine movement [34], it is hypothesized that skepticism and hesitancy will be observed more among the far ends of the political and religious spectrum—the most politically and religiously conservative Americans, as well as the most politically progressive secularists—with the highest rate of compliance in between these two poles. It is also hypothesized that skepticism and hesitancy will be greatest among those of lower socioeconomic standing in terms of financial resources and education. To test these expectations, data will be analyzed from a newly released national probability survey of the adult population of the U.S.

## Methods

This study analyzed data from the sixth round of the Values and Beliefs of the American Public Survey (VBAPS), a stratified random probability sample of 1248 U.S. adults ages 18 and older living in all 50 states and D.C. The survey was conducted by the Gallup Organization in accordance with all relevant guidelines and regulations. Data were collected from January 27 to March 21, 2021, using mail and web surveys (AAPOR1 response rate = 11.3%). Weights were included to adjust the sample to known demographic characteristics of the U.S. adult population by geographic Census region, age, gender, race/ethnicity, and education based on the 2020 Current Population Survey [35]. Many of the measures in the VBAPS are not available in other national surveys, so this is a new and unique source of information on determinants of COVID-19 beliefs and attitudes.

Outcome measures for the present analyses were *COVID-19 skepticism* (“The dangers of the COVID-19 pandemic are exaggerated by mainstream media”) and *SARS-CoV-2 vaccine hesitancy* (“A vaccine for COVID-19 should not be trusted”), with response categories for both ranging from 1 = strongly disagree to 5 = strongly agree. For purposes of this study, binary variables were created for strongly agree or agree (coded 1) compared with strongly disagree, disagree, or neither agree nor disagree (coded 0). Agreement was estimated across categories of several exposure variables, including both political and religious measures. Political variables used in the present analyses were *political party identity* (coded 1 = strong Democrat to 7 = strong Republican), *political orientation* (coded 1 = extremely liberal to 7 = extremely conservative), and *Presidential voting preference* (dummy variables for wanting Biden, Trump, or some other candidate to win). Religious variables assessed *Bible beliefs* (“The Bible means exactly what it says. It should be taken literally, word-for-word, on all subjects,” compared with three other categories of personal beliefs about the Bible) and *belief in God* (a series of dummy variables indicating “no doubts,” belief in a “higher power or cosmic force,” and a few response categories collapsed into agnostic and atheist). Percentages of respondents who agreed with the skepticism and hesitancy items were also estimated separately by categories of gender, marital status, race/ethnicity, age, urbanicity, region, education, annual family income, and whether the respondent had “been infected by COVID-19” or had “lost a close relative or friend to COVID-19.”

All analyses were conducted using Stata 15. Prevalence rates were estimated separately for COVID-19 skepticism and SARS-CoV-2 vaccine hesitancy by categories of the political and religious variables and by sociodemographic categories, using complete data for each bivariate association. A series of multivariable models was then estimated using binary logistic regression, with multiple imputation for missing cases [36], a standard procedure for epidemiologic and population-health research [37]. Dependent variables were used in the imputation process, but their imputed values were deleted prior to estimating the models. Results are based on five imputed datasets, and were comparable when listwise deletion was employed and when additional imputed datasets were analyzed. The final imputed sample used for the logistic regression analyses contained 1222 respondents. Findings are reported as prevalence odds ratios with associated 95% confidence intervals.

## Results

**Fig. 1:**
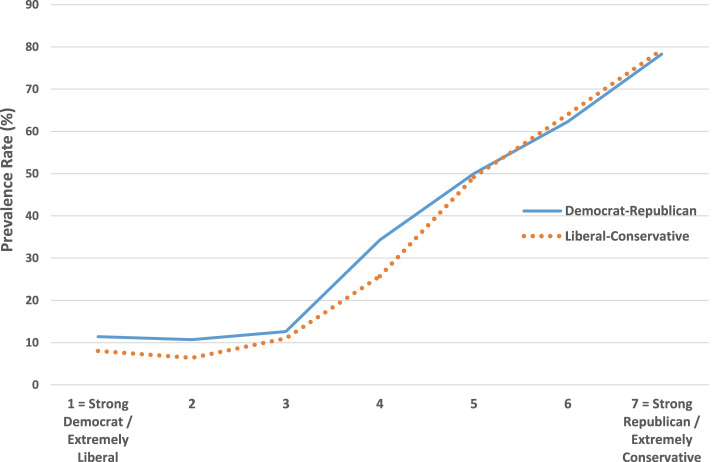
COVID-19 skepticism by political party identity and political orientation

**Fig. 2:**
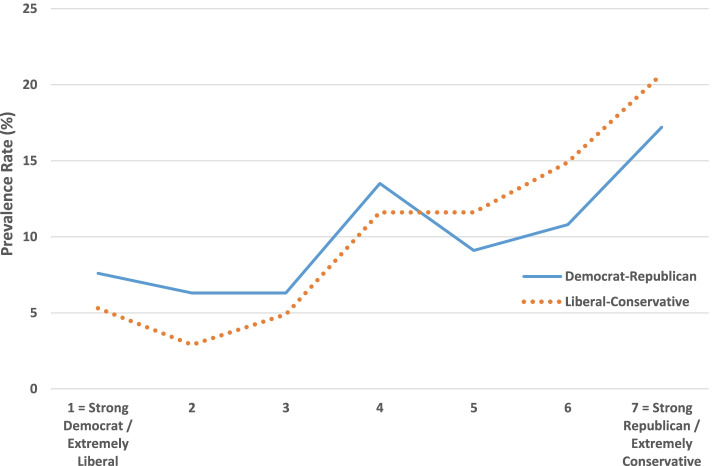
SARS-CoV-2 vaccine hesitancy by political party identity and political orientation

Table 1 shows that COVID-19 skepticism and SARS-CoV-2 vaccine hesitancy are strongly associated with Republican and conservative political preference, in an almost linear fashion (Figs. 1 and 2), and similarly with conservative religious beliefs about the Bible and God. The prevalence of skepticism among Trump voters (69.3%) compared to Biden voters (12.0%) is especially pronounced. Sociodemographic determinants are less clear-cut: minimal differences across most categories except for substantially greater skepticism among males, married individuals, non-Black people, the rural population, and those without a graduate degree, and greater vaccine hesitancy among Black and Hispanic people, the rural population, Southerners, individuals with a high school education or less, and those in the lowest categories of family income. For education and income, there are gradients with hesitancy but mostly not with skepticism, except for less skepticism among those with college or a graduate degree. A history of COVID-19, in oneself or a family member or close friend, does not predispose for skepticism, but modestly so for hesitancy.

**Table 1:** Prevalence of COVID-19 skepticism and SARS-CoV-2 vaccine hesitancy in the U.S., by political, religious, and sociodemographic categories: January–March, 2021

	COVID-19 Skepticism (% agree or strongly agree)		SARS-CoV-2 Vaccine Hesitancy (% agree or strongly agree)	
Exposure variables	%	(n)	%	(n)
Political party identity				
1 = Strong Democrat	11.4	(211)	7.6	(210)
2	10.7	(206)	6.3	(206)
3	12.6	(127)	6.3	(127)
4	34.3	(350)	13.5	(349)
5	50.0	(110)	9.1	(110)
6	62.3	(151)	10.8	(148)
7 = Strong Republican	78.3	(92)	17.2	(93)
Political orientation				
1 = Extremely liberal	8.0	(75)	5.3	(75)
2	6.4	(203)	2.9	(204)
3	11.0	(146)	4.9	(144)
4	25.7	(389)	11.6	(387)
5	49.2	(128)	11.6	(129)
6	64.0	(250)	14.9	(249)
7 = Extremely conservative	79.3	(58)	20.7	(58)
Presidential voting preference				
Biden	12.0	(725)	7.2	(723)
Other	38.3	(128)	13.3	(128)
Trump	69.3	(381)	14.8	(379)
Bible beliefs				
Bible is an ancient book	16.4	(324)	4.6	(323)
Bible contains human error	30.9	(162)	12.4	(161)
Bible is true but not literal	39.7	(370)	10.8	(370)
Biblical literalist	50.8	(183)	17.9	(184)
Belief in God				
Atheist	8.3	(84)	1.2	(84)
Agnostic	27.8	(281)	8.2	(280)
Belief in a higher power	23.3	(189)	9.6	(188)
No doubt God exists	41.5	(595)	13.3	(593)
Gender				
Female	27.3	(656)	11.8	(653)
Male	38.2	(555)	9.2	(553)
Marital status				
Married	36.8	(646)	9.2	(644)
Not married	27.6	(576)	12.0	(574)
Race/ethnicity				
White	33.9	(799)	7.9	(800)
Black	22.1	(136)	19.4	(134)
Hispanic	32.1	(190)	16.9	(189)
Other	35.2	(88)	9.3	(86)
Age				
< 65 years old	33.6	(803)	11.6	(800)
> 65 years old	30.4	(470)	8.3	(468)
Urbanicity				
City	25.9	(305)	10.9	(303)
Suburb	26.9	(346)	7.8	(345)
Small town	35.1	(388)	11.1	(386)
Rural	48.8	(172)	15.1	(172)
Region				
Northeast	30.6	(209)	7.6	(210)
South	33.8	(477)	13.8	(472)
Midwest	32.2	(270)	10.7	(270)
West	32.6	(310)	7.1	(309)
Education				
< High school	37.0	(46)	18.8	(48)
High school	41.6	(125)	17.9	(123)
Some college	38.3	(428)	11.7	(428)
College degree	30.6	(346)	9.4	(342)
Graduate degree	18.2	(258)	4.7	(257)
Annual family income				
< $10,000	32.3	(65)	21.5	(65)
$10,001 to $20,000	31.1	(106)	19.0	(105)
$20,001 to $35,000	36.4	(151)	13.8	(152)
$35,001 to $50,000	29.6	(179)	11.4	(175)
$50,001 to $100,000	33.8	(320)	11.3	(320)
$100,001 to $150,000	29.6	(189)	3.7	(189)
> $150,001	31.4	(185)	3.3	(184)
COVID-19 exposure				
Has been infected	36.3	(215)	14.0	(215)
Has not been infected	31.6	(1087)	9.7	(1032)
COVID-19 family fatality				
Lost close relative or friend	31.3	(316)	14.7	(314)
Did not lose relative or friend	32.9	(938)	9.0	(935)

In Table 2, for skepticism, odds ratios for the political and religious variables remained statistically significant (i.e., 95% confidence intervals did not include 1.0) even after adjusting for effects of all of the sociodemographic variables. For vaccine hesitancy, this was true for the political but not the religious indicators. For both outcome variables, significantly higher adjusted odds were observed for Republican party identity and conservative political orientation. In other words, the stronger one’s affirmation of each of these two political constructs, the greater the odds of skepticism and hesitancy.

**Table 2:** Binary logistic Regressions of COVID-19 skepticism and SARS-CoV-2 vaccine hesitancy on political and religious exposure variables: unadjusted and adjusted models

	**COVID-19 Skepticism**							
**Exposure variables**^**a,b**^	**OR**^**c**^	**(C.I.)**^**c**^	**OR**	**(C.I.)**	**OR**	**(C.I.)**	**OR**	**(C.I.)**
Republican party	1.9 1.9	(1.7, 2.1) (1.7, 2.1)						
Conservative politics			2.2 2.2	(1.9, 2.5) (1.9, 2.5)				
Biblical literalist					2.9 2.7	(1.9, 4.4) (1.7, 4.2)		
No doubt God exists							2.4 2.4	(1.7, 3.5) (1.6, 3.6)
	**SARS CoV-2 Vaccine Hesitancy**							
**Exposure variables**^**a,b**^	**OR**^**c**^	**(C.I.)**^**c**^	**OR**	**(C.I.)**	**OR**	**(C.I.)**	**OR**	**(C.I.)**
Republican party	1.2 1.4	(1.1, 1.4) (1.2, 1.6)						
Conservative politics			1.4 1.5	(1.2, 1.6) (1.3, 1.7)				
Biblical literalist					1.8 1.3	(1.0, 3.3) (0.7, 2.5)		
No doubt God exists							1.7 1.4	(1.0, 2.8) (0.8, 2.5)

*Note*. *N* = 1215

^a^Each of the four exposure variables is included separately in its own models. In each cell, unadjusted (bivariate) results are listed above adjusted (multivariable) results

^b^All adjusted analyses control for effects of gender, marital status, race/ethnicity, age, urbanicity, region, education, and annual family income

^c^Cell entries are prevalence odds ratios from respective binary logistic regressions, with 95% confidence intervals listed in parentheses

In additional results (not reported in Table 2), stratifying by whether or not one had been infected with the SARS-CoV-2 virus or had lost a relative or close friend to COVID-19 did not substantively alter the results for skepticism for either political variable. For vaccine hesitancy, having been infected with SARS-CoV-2 modestly reduced the odds due to Republican (OR = 1.3, C.I. = 1.0–1.7) and conservative (OR = 1.3, C.I. = 1.0–1.8) preference; having lost someone close did likewise for Republican (OR = 1.1, C.I. = 0.9–1.4) and conservative (OR = 1.1, C.I. = 0.9–1.5) preference.

## Discussion

As seen in these analyses, our hypotheses were half right and half wrong. In these data, COVID-19 skepticism and SARS-CoV-2 vaccine hesitancy do not appear to be phenomena of both poles of the political and religious spectrum, as anticipated, but largely products of identification with the political and religious right. This finding is consistent with results of a recent online study [38], with European data [39], and with a U.S. Census Bureau household survey [40]. The findings for U.S. Presidential voting preference were, frankly, stark, though perhaps not unexpected [41]. The results for education and income suggest that skepticism and hesitancy may not be entirely a matter of lack of knowledge or lack of resources, and thus the solution may not be primarily about more health education or better access to vaccines. The problem instead may be philosophical and ideological and perhaps this is why the unvaccinated have proven so intransigent [42]. Note also that, in absolute numbers, skepticism does not inherently translate into vaccine hesitancy, but prevalences of the latter are still suboptimal for ending the pandemic. Still, the two issues are not as linked as one might have expected.

Up to now, exposing the myths inherent in skepticism and hesitancy has been ineffective in countering resistance to immunization. Noncompliance with primary-preventive measures remains “a significant impediment to suppression of SARS-CoV-2 spread” and thus requires more creative approaches [43]. For example, providing evidence of the dangers of communicable disease exposure to unvaccinated individuals, especially vulnerable loved ones such as children, was found to be a better strategy to combat antivaccination attitudes pre-COVID-19 [44]. People are jealous of their beliefs and ideologies, but, one hopes, are more jealous of the well-being of their family members. Regardless, as has been observed since early in the vaccine rollout, efforts to address the persistent lacuna of immunization have met with strident pushback, motivated in part by political and religious zealousness [45].

The observation that personal experience with COVID-19, in oneself or a loved one, did not mitigate the effect of politics on skepticism and only barely reduced its greater odds for hesitancy should raise alarms. The expectation that both COVID-19 skepticism and SARS-CoV-2 vaccine hesitancy will fade as more and more people, or those whom they know, fall victim to the disease may not be accurate. Nor are opinion leaders as significant here as might be hoped. One should recall that in August, 2021, speaking at rally of supporters, when former President Trump implored the crowed to get vaccinated he was met with “booing and jeering” [46]. For good reason, many epidemiologists and physicians are pessimistic about immunization coverage ever reaching a level that will end the pandemic through attaining herd immunity [47, 48], although so far this sentiment is not unanimous among biomedical scientists [49].

## Conclusion

As noted, efforts to increase immunization in the U.S. through public education may be inadequate; resistance appears ideological, not primarily the result of lack of access to accurate information. Nor is it clear that additional federal expenditures to facilitate increased access to vaccines would be money well spent. While there is an observable prevalence gradient with income, even in the lowest income categories the rate of hesitancy is not much higher than that of the most politically conservative respondents or of Biblical literalists. Moreover, according to our findings, adjusting for the effects of income did not reduce the greater odds due to political preference. Lack of financial resources may not be the overriding barrier to vaccine access here as others have concluded [50], although it is surely a co-factor. Other solutions therefore may need to be considered, including broader government mandates, which at the time of this writing have been implemented in places and been met with widespread political resistance [51], in some instances violent [52]. The alternative is to stand by while new variants have the opportunity to emerge, adding to the increasing fatality count and continuing to overburden a medical care system that has already found itself at the breaking point multiple times during the pandemic.

Unless and until the immunization rate increases very substantially—and at present that does not appear likely in the near term—the COVID-19 pandemic may continue to persist until enough people are exposed to and infected by SARS-CoV-2 that they either acquire lasting immunity or are culled from the population in numbers that expand the fatality count far past where it is at present. The ideological roots of the present crisis of skepticism and hesitancy appear to be a downstream legacy of decision-making early in the pandemic that was motivated by political as well as scientific considerations, and the results presented here suggest that the challenges being faced may not be close to resolving.

## Data Availability

The data source used for the current study is a national survey conducted by the Gallup Organization. More information on the survey, including contact information and accessibility can be found at https://www.baylor.edu/baylorreligionsurvey/ (Dr. Paul Froese, director).
